# Sex Differences in Bone-Active Medication Utilization Before and During the Year After Hip Fracture

**DOI:** 10.1093/geroni/igaa057.719

**Published:** 2020-12-16

**Authors:** Jennifer Kirk, Denise Orwig, Ann Gruber-Baldini, Marc Hochberg, Jay Magaziner, Alan Rathbun

**Affiliations:** 1 University of Maryland Baltimore, Laurel, Maryland, United States; 2 Division of Gerontology, Baltimore, Maryland, United States; 3 University of Maryland School of Medicine, Baltimore, Maryland, United States; 4 University of Maryland, Baltimore, Baltimore, Maryland, United States

## Abstract

Bone-active medications (BAM) [prescriptions (RxBAM) and supplements(calcium/vitamin D] increase bone mineral density and reduce osteoporotic fracture risk. However, RxBAM utilization rates are low, and it is unclear who is treated with BAMs before/after a hip fracture. This study examined sex differences in BAM use at baseline and predicted the probability of RxBAM use during follow-up(2, 6, and 12-months). The sample included frequency-matched males and females 65 years or older from the Baltimore Hip Studies’ seventh cohort. Differences in baseline characteristics between males and females with complete data(n=313) were assessed using t-tests and chi-square tests. Generalized estimating equations(GEE) predicted the probability of RxBAM use by sex among participants(n=270) with outcome data during follow-up adjusted for baseline characteristics. Prior to fracture, there were sex-differences in BAM use, with fewer men than women taking RxBAMs(9% versus 26%), calcium(18% vs. 57%) and vitamin D (55% vs. 68%). These differences remained over the year post-hip fracture. Only 12(3.5%) participants took RxBAM the entire study period. Of RxBAM users n=70(26%), there were few new-users (n=35), and many participants stopped or never started treatment. Unadjusted GEEs showed that men were less likely to use RxBAM (OR= 0.42; 95% CI:0.22,0.78, p=.007), during the hip fracture recovery period compared to females. However, after controlling for differences in baseline characteristics between males and females, particularly pre-fracture BAM medication use, the observed association (OR=0.62; 95% CI:0.29, 1.31; p= 0.23). RxBAM use was low, especially in men and may contribute to the high rates of preventable subsequent osteoporotic fractures and post-fracture mortality.

